# The Biochemistry and Effectiveness of Antioxidants in Food, Fruits, and Marine Algae

**DOI:** 10.3390/antiox12040860

**Published:** 2023-04-02

**Authors:** Lavinia Lorena Pruteanu, David Stanley Bailey, Andrei Cristian Grădinaru, Lorentz Jäntschi

**Affiliations:** 1 Department of Chemistry and Biology, North University Center at Baia Mare, Technical University of Cluj-Napoca, 430122 Baia Mare, Romania; 2 Research Center for Functional Genomics, Biomedicine and Translational Medicine, “Iuliu Hațieganu” University of Medicine and Pharmacy, 400337 Cluj-Napoca, Romania; 3 IOTA Pharmaceuticals Ltd., St Johns Innovation Centre, Cowley Road, Cambridge CB4 0WS, UK; 4 Department of Genetics, Faculty of Veterinary Medicine, “Ion Ionescu de la Brad” University of Life Sciences of Iaşi, 700490 Iaşi, Romania; 5 Institute of Doctoral Studies, Babeş-Bolyai University, 400084 Cluj-Napoca, Romania; 6 Department of Physics and Chemistry, Technical University of Cluj-Napoca, 400114 Cluj-Napoca, Romania

**Keywords:** biochemistry, health, antioxidants, free radicals, fruits, vitamin C, food, marine algae, food additives

## Abstract

It is more effective to maintain good health than to regain it after losing it. This work focuses on the biochemical defense mechanisms against free radicals and their role in building and maintaining antioxidant shields, aiming to show how to balance, as much as possible, the situations in which we are exposed to free radicals. To achieve this aim, foods, fruits, and marine algae with a high antioxidant content should constitute the basis of nutritional elements, since natural products are known to have significantly greater assimilation efficiency. This review also gives the perspective in which the use of antioxidants can extend the life of food products, by protecting them from damage caused by oxidation as well as their use as food additives.

## 1. Introduction

Antioxidants are a class of a multitude of chemical substances clearly associated with large health benefits and lower risks of various age-related diseases.

**Figure 1:**
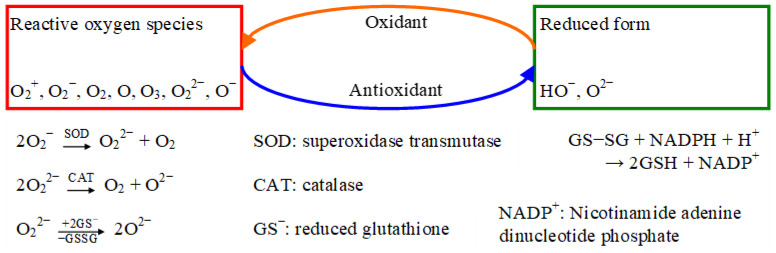
Biochemistry of antioxidants (formation and equilibrium reactions in Ref. [4]; oxyl radical detection in [5]; antioxidants reduction in [6]).

They also can stop the damaging actions of reactive oxygen species (ROS) [1,2], which include partially reduced or “energized” forms of oxygen, some of them as “free radicals”, with an unpaired electron included in an orbital, while others as “nonradical species”, such as hydrogen peroxide and singlet oxygen, whose reactivity is even greater than that of the ground state of molecular oxygen [1,3]. A schematic way of antioxidants action neutralizing free radicals by reacting together is shown in Figure 1, adapted from [2,4,5,6].

**Figure 2:**
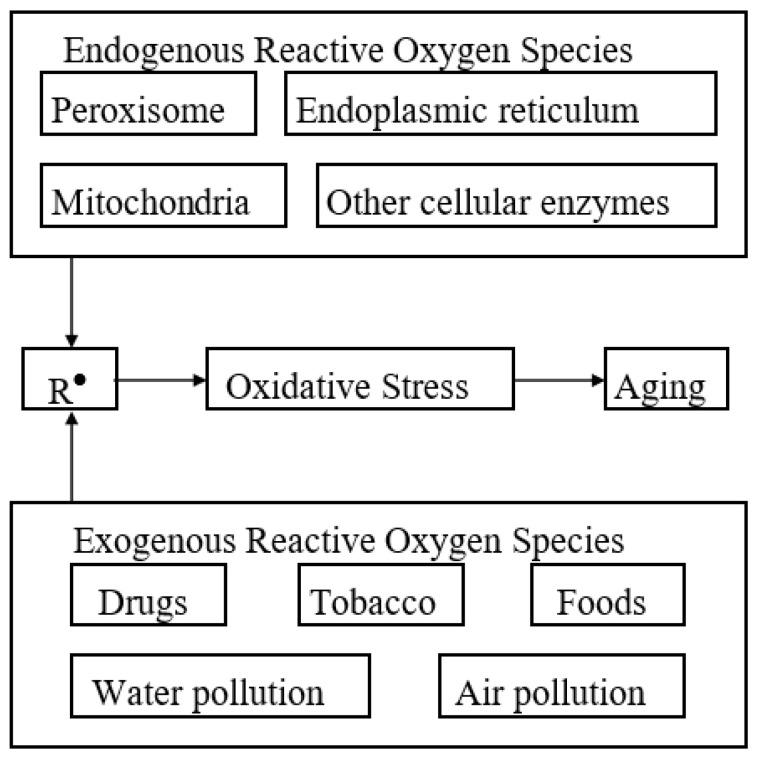
Endogenous and exogenous sources contributing to aging (free radicals are the leverage in oxidative stress and aging).

**Figure 3:**
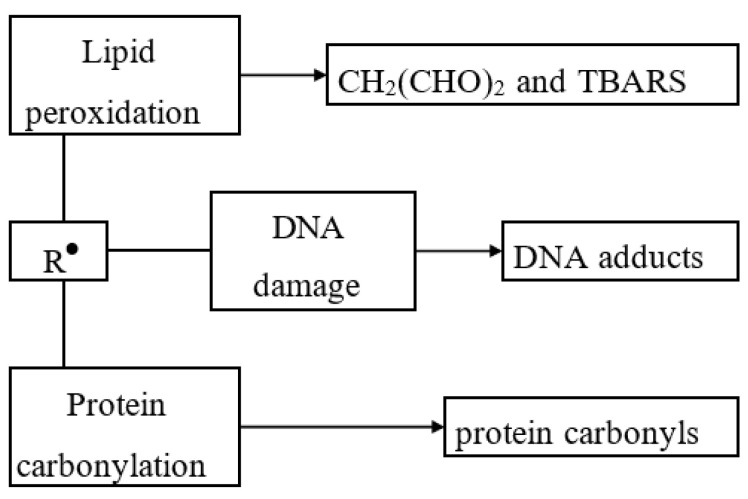
Damaging actions of free radicals (most organic radicals have short lifetimes; many radicals spontaneously dimerize; however, during their short lifetime, due to the presence of unpaired electrons, the radicals are highly chemically reactive and may damage the biological tissue). TBARS, thiobarbituric acid reactive substance (formed as a result of lipid peroxidation).

Endogenous and exogenous sources of free radicals are presented in Figure 2, while some of their damaging actions are schematically presented in Figure 3 (adapted from [7]).

ROS are produced by normal aerobic metabolism, via environmental factors, such as smoke and radiation, an excess of drugs, or an incorrect nutritional style [8,9,10]. Free radicals damage nuclear DNA, proteins, and the lipid matrix of cells [11,12,13]. Once they enter the body, they not only cause aberrant cell development but can also cause genetic changes that are the basis of relentless disease [14,15].

**Figure 4:**
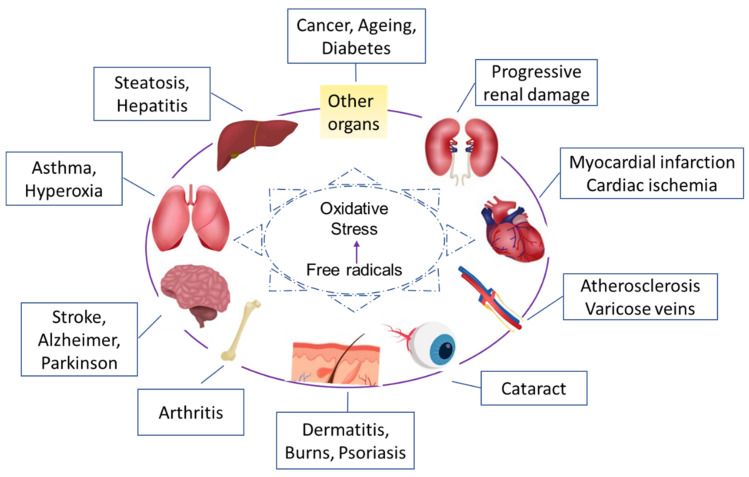
Most frequent pathologies related to oxidative stress.

Studies have shown that reactive oxygen molecules are involved in more than 50 medical conditions, including various forms of cancer [16], heart disease [17,18], premature aging [19], cataracts [20], and even AIDS [21], or in pregnancy [22] (Figure 4).

It is estimated that a single cell is exposed to free radical damage 10,000 times a day [23]. Many of the resulting injuries are repaired by the body, but some accumulate. Some researchers claim that aging is due to the accumulation during the life of unrepaired damage to the deoxyribonucleic acid inside the mitochondria, damage caused mostly by the action of free radicals [24,25]. Free radicals are usually destroyed by our body’s natural antioxidant system [26]. There are both stable and unstable molecules of oxygen in the body: While stable oxygen is essential for sustaining life, unstable oxygen molecules (free radicals) can also be useful because they can be harnessed to fight inflammation, and bacteria, and control muscle tone, regulating the functioning of internal organs and blood vessels [26,27].

The problem with free radicals lies in their imbalance, their regulation often being compromised [28]. Many of the body’s natural biological processes, such as breathing, digesting food, neutralizing alcohol, and drugs, and converting fat into energy produce harmful free radicals [8,9,10]. These free radicals can trigger a negative chain reaction in the body, a reaction that destroys the cell membrane, blocks the action of the main enzymes, prevents cellular processes and normal cell division, destroys cellular DNA, and blocks energy generation [26,27,28].

Understanding these mechanisms and their biochemistry at the molecular level allows us to further explain and emphasize the importance of antioxidants intake for combating the negative effects of ROS, which belong to the category of oxidants. With this purpose, in our review, we elaborate on these causes and effects situations (ROS vs. antioxidants) and the health benefits of these. In this regard, we will further present in the next sections, numerous studies that have highlighted the importance of a diet rich in antioxidant compounds (i.e., polyphenols, thiols, vitamins C and E, as well as some minerals) for the prevention of various chronic-degenerative diseases related to an increase in oxidative stress, caused by free radicals, but also we will present the aspects of promising studies currently elaborated for delivering new resources of antioxidants, such as the marine extracts.

More than this, another important aspect that we considered in this review is to clarify the antioxidants terminology, since this refers to multiple sides and has different meanings in food and health science as follows [29].

## 2. Biochemistry of Antioxidants and Their Mode of Action

Endogenous antioxidants are body products. In contrast with the exogenous antioxidants, the body possesses enzyme systems with an antioxidant action (superoxide dismutase, glutathione peroxidase, and catalysis), co-participating in the deactivation of some free radicals that are formed in the body [30,31]. As a defense against oxidative stress imbalances, the body has produced so-called endogenous antioxidants, enzyme systems capable of annihilating free oxygen molecules, preventing the production of negative effects in the body [32,33]. Among the endogenous antioxidants, we mention superoxide-dismutase, catalase, glutathione peroxidase, and hydropersulfides [34,35]. Some subtypes of glutathione peroxidase are selenium (Se)-dependent, and recent studies [36,37,38,39,40] show that an increased intake of Se is associated with protection against the development of cancer and other chronic diseases.

Antioxidants as food additives are referring to some natural or synthetic (established) antioxidants which are also widely used in the food industry to prevent a reduction in the oxidation of fats or other components present in food, during the preservation period [41,42].

Exogenous antioxidants are introduced with food and are referred to the established or natural antioxidants. Because it is much more effective, and cheaper, to maintain good health rather than to regain it, the best protection against free radicals is to build and maintain “antioxidant shields”, through a regimen of adequate food with little fat, rich in digestive fibers, and in antioxidant substances, such as vitamin E, vitamin C, and beta-carotene, combined with regular exercise [43,44], and through a life program [45] aimed at avoiding, as much as possible, the situations in which we are exposed to the attack of free radicals. Foods with a high content of antioxidants constitute the basis of nutritional strategies that we can take from external sources [46,47]. Food of plant origin was associated with a high content of antioxidants [47,48]. Importantly, exogenous antioxidants that can be taken in the diet have the same role in reducing the excessive number of free radicals. The most important of these external (or exogenous) antioxidants are vitamin C, vitamin E, and beta-carotene [49].

**Figure 5:**
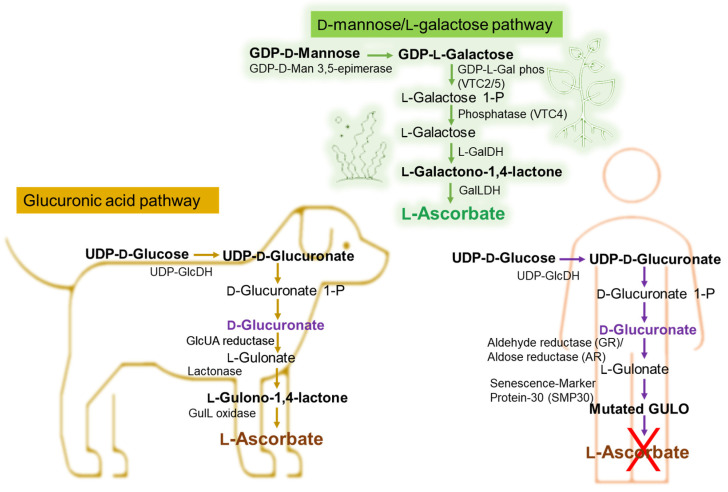
Biochemical pathway of vitamin C synthesis in animals vs. plants/green algae and humans. GDP-L-Gal phos, guanosine diphosphate-L-galactose phosphorylase; GalDH, galactose dehydrogenase; UDP-GlcDH, uridine diphosphate glucose dehydrogenase; GlcUA reductase, glucuronic acid reductase; GulL oxidase (GULO), L-gulonolactone oxidase. This figure is based on information from references [61,62,63,64,65].

Taking the example of vitamin C’s different pathways to biosynthesis in marine algae or plants, animals, and the human bodies, we can emphasize the importance of the exogenous addition of antioxidants such as vitamin C in the human body. The Smirnoff–Wheeler pathway, in which vitamin C is synthesized from D-mannose and L-galactose (D-mannose/L-galactose pathway) [50], represents the major route of vitamin C biosynthesis in marine algae and plants, at the cellular level (Figure 5), the other three involved routes being the glucose, myoinositol, and the galacturonate pathways [51,52,53,54]. Most animals produce relatively high levels of ascorbic acid from glucose in the liver via the glucuronic acid pathway (Figure 5) [55,56,57,58]. Humans are unable to synthesize vitamin C and must ingest this vitamin [59,60].

As shown in Figure 5, in certain vertebrates (i.e., dogs), L-Ascorbate synthesis involves three enzymatic steps starting from the conversion of D-Glucuronate, with L-Gulonate and L-Gulono-γ-lactone (L-Gulono-1,4-lactone) as intermediate metabolites [61,62,63,64,65,66]. If in this case, the final enzymatic step is catalyzed by L-Gulono-γ-lactone oxidase (GULO), converting L-Gulonate to L-Ascorbate, in humans, the GULO enzyme is mutated (Figure 5) and not functional in primates also including guinea pigs and some spontaneous mutant mouse and rat models [65,67]. Instead, the conversion of L-Gulonate to L-Gulono-1,4-lactone occurs via Senescence-Marker Protein-30 (SMP30) (Figure 5) also known as regucalcin [62,65]. Likewise, in the humans’ case, the conversion of D-Glucuronate to L-Gulonate occurs mainly through aldehyde reductase (GR) and to a smaller extent with aldose reductase’ (AR) contribution (Figure 5) [51,65,66]. Considering that the common molecular mechanism of the body’s limited ability to synthesize vitamin C is the absence of GULO [68,69,70,71], genetically, it is considered that the loss of synthesizing the ascorbic acid is likely due to the complete loss of the L-gulono-γ-lactone oxidase (GULO) gene.

Combinations of all these facts bring us to the conclusion of an important balanced diet associated with antioxidant supplements, potentiating each other’s effects and influencing the prevention of diseases, such as heart disease, arthritis, visual impairment, stroke, and premature aging of the skin, enhancing well-being.

Membrane lipids represent a major target of the radical attack, due to the presence of double bonds in the structures of the polyunsaturated fatty acids which comprise them. Membrane phospholipids most frequently contain unsaturated fatty acids, i.e., linoleic acid, linolenic acid, and arachidonic acid [27,28]. Membrane lipid peroxidation affects the structure and functions of the plasma membrane and the membranes of intracytoplasmic organelles so that transmembrane potentials, ion fluxes, and transmembrane transport are disturbed, and membrane receptors are inactivated and signaling pathways are deregulated [71,72].

The process of lipid peroxidation changes not only the lipid components of membranes but also the proteins, following the reaction of some amino acids with the aldehyde products of peroxidation [71]. Oxidative changes in proteins under the action of reactive oxygen species can also cause the inactivation of enzymes and membrane proteins [73], thereby producing structural changes that lead to the destabilization of cell morphology. The products generated because of lipid peroxidation are involved in inflammatory diseases [74], aging [75], hepatotoxicity [76], hemolysis [77], and all phases of carcinogenesis during the appearance of malignant tumors and metastases [78].

The effect of reactive oxygen species on enzymes includes, for the most part, decreased catalytic capacity, often caused by the oxidation of sulfhydryl groups and the modification of amino groups [79,80]. Some free radicals result from normal cellular processes, for example, when cells use oxygen as fuel for energy production, free radicals appear as secondary products of this metabolic process necessary to sustain life [9]. On the other hand, both the environment, in which we live, and the living environment are other main factors causing reactive oxygen species [9,12]. Antioxidants can interrupt the sequence of oxidation reactions before it is initiated. In general, antioxidants have a high reduction potential, releasing hydrogen ions, with the inhibition process proceeding as shown in the following representation (Equation (1)) [81]:

InH + RO_2_^−^ → RO_2_H + In^−^ (1)

where InH is an antioxidant, RO^−^_2_ is a free hydroperoxide radical ion, RO_2_H is hydroperoxide of, e.g., a fatty acid, and In^−^ is an inactive or weakly active radical ion.

But in all cases, with the increase in the inactivation duration, there is a decrease in the number of antioxidants—the increase in the peroxide index is found only after there has been a significant decrease in the added antioxidant [2,82,83]. Taking here the α-tocopherol (Vitamin E) as an example of lipid-soluble antioxidant, which acts as a “chain breakerf” to intercept lipid peroxyl radicals (LOO˙) and to terminate the lipid peroxidation chain reactions (Equation (2)) [79], it can be seen that the mechanism of action is much more complex, as the antioxidants can act at successive steps of initiation, propagation, and chain termination of the oxidative radical process [80].

OO˙ + α-tocopherol–OH → LOOH + α-tocopherol–O˙(2)

It can be explained that there is a close correlation between the structure of antioxidants and their mode of action, determined by factors as follows [84,85]:

- The presence of the aromatic nucleus of phenol or naphthol, of a secondary or tertiary hydroxyl group which increases the effectiveness (most antioxidants have a phenolic structure).
- The presence of allylic groups in the ortho or para position compared to the hydroxyl groups which have a favorable effect.
- The antioxidant effect increases proportionally with the length of the chain.
- Alkylation in the meta position is less effective.
- The esterification of the hydroxyl groups which causes a total disappearance of the antioxidant activity.

A classification of antioxidants according to their mode of action is presented in Table 1 [83,86].

Glutathione peroxidase (GSHPx), catalase (CAT), and superoxide dismutase (SOD) (mentioned in Figure 1) act as the first-line defense antioxidants, as their importance is especially related to superoxide anion radical (*O_2_) which is perpetually generated in normal body metabolism, particularly through the mitochondrial energy production pathway (MEPP) and their fundamental role in preventing oxidative stress and the cellular damage [31,79,82].

*Glutathione* is a nonenzymatic antioxidant that is found in most cells, and tissues of plants and animals, and in humans, the highest levels are in the liver, lens, pancreas, spleen, and kidney [87,88]. It is mainly synthesized by the body [88], and it can increase the level of cytotoxic T cells in lymphocytes and neutralize free radicals [89,90]. Given that glutathione has a tripeptide composition of cysteine, glutamate, and glycine, it has an active thiol (SH−) within the cysteine structure [32,34]. In the cell, >98% of glutathione is found in the reduced thiol form (GSH) [31,88], but due to the cysteine residues that can be easily oxidized nonenzymatically by various electrophilic substances (free radicals, reactive oxygen, and nitrogen species), it is also present in the oxidized form as glutathione disulfide (GSSG) or glutathione peroxidase [31,88]. After synthesis, it is distributed to intracellular compartments and the extracellular space for use by other cells and tissues [88,90]. The rate of GSH synthesis is largely controlled by the degree of expression and catalytic activity of the enzyme γ-glutamyl-cysteine synthetase (GCS) and the cellular availability of cysteine [31,82,88,89,90]. Oxidative stress, inflammatory cytokines, cancer, chemotherapy, ionizing radiation, heat shock, inhibition of GCS activity, GSH depletion, GSH conjugation, heavy metals, antioxidants, and insulin increase γ-glutamyl-cysteine synthase transcription or activity in a wide variety of cells [91,92]. In contrast, protein deficiency, dexamethasone, erythropoietin, TNF-β (tumor necrosis factor), hyperglycemia, and GCS phosphorylation decrease GCS transcription or activity [91,93,94]. The glutathione system also represents a “capture system” for peroxides from water metabolism and lipid peroxides permanently formed in the cell, metabolizing them with the formation of water and oxygen [31,88,89,90]. It provides important protection for the mitochondrial and cell membrane against the harmful effects of reactive oxygen species (oxidative stress) [31], protects the tertiary structure of proteins, and activates the transport of amino acids through the cell membrane [31,95]. The cellular level of glutathione is stimulated by alpha lipoic acid, glutamine, colostrum, selenium, and vitamins C, B6, and B2, and the effectiveness of vitamins C, E, and coenzyme Q10 depends on the level of glutathione in the body [96]. Food sources rich in GSH are generally green leafy vegetables, such as spinach, parsley, and broccoli. However, glutathione from food is only partially absorbed, being mostly hydrolyzed by peptidases [96,97,98]. However, the diet plays an important role in the exogenous intake of glutathione by providing important cofactors, such as Se, Mn, Zn, and S-containing amino acids. GSH has a dual role in our health and pathology as an antioxidant and in the detoxification of certain xenobiotics [96,97,98].

*Catalases*. While GSHPxs are cytosolic residents, catalases are mainly found in peroxisomes, in the liver, and erythrocytes, but some catalases are found in all tissues [82,99], being the first characterized antioxidant enzymes [88] and being one of the crucial antioxidant enzymes that mitigate oxidative stress to a considerable extent by destroying cellular hydrogen peroxide to produce water and oxygen by using either iron or manganese as a cofactor [31,100]. Basically, they are present in almost all living tissues that utilize oxygen [31]. Due to its chemical structure of four subunits, each containing a heme group and a molecule of NADPH, catalase basically catalyzes the conversion of hydrogen peroxide to water and oxygen [82], while superoxide dismutase (one of the most potent intracellular enzymatic antioxidants) catalyzes the conversion of superoxide anions to dioxygen and hydrogen peroxide [82]. Hence, all three, catalase, glutathione peroxidase, and superoxide dismutase, are functionally interconnected due to the hydrogen peroxide (H_2_O_2_), which is produced as a result of the reaction catalyzed by SOD, H_2_O_2_ being the substrate of both CAT and GSHPx [87]. Deficiency or malfunction of catalase causes aging disorders and pathogenesis of degenerative diseases, such as diabetes mellitus, hypertension, anemia, vitiligo, Alzheimer’s disease, Parkinson’s disease, bipolar disorder, cancer, schizophrenia, or even male infertility [100,101]. 

*Superoxide dismutase*. Depending on its expressed activity, superoxide dismutase may act either as an antioxidant or as a prooxidant [88] as exists in several isoforms, differing in the active metal center, amino acid composition, cofactors, and other properties [82], and neutralizes superoxide ions by going through successive oxidative and reductive cycles of transition metal ions at its active site [82]. In humans, three forms of SOD are present: cytosolic Cu, Zn-SOD (consisting of a dinuclear metal cluster with copper and zinc ions, which catalyzes the dismutation of the superoxide anion to oxygen and water), the mitochondrial Mn-SOD (a homotetramer that includes one manganese atom per subunit, which partitions the superoxide anion), and the extracellular superoxide dismutase containing copper and zinc (a tetrameric secretary glycoprotein having a high affinity for certain glycosaminoglycans) [82,102,103]. In contrast with the fact that superoxide dismutase is indispensable to cellular health, that is protecting body cells from oxidative stress, and that helps in the process of aging or cell death, superoxide dismutase enzyme deficiency is quite common, and more than this, levels of superoxide dismutase decline with age, whereas free radical formation increases [31]. As a result, plant sources of SOD and SOD supplementation became of interest for health enhancement [103,104]. It has been reported that a considerable and adequate daily SOD supplementation protects the immune system and significantly reduces the chances of degenerative diseases and aging pathogenesis, and there are several natural resources that can assure the daily intake of SOD, such as cabbage, Brussels sprouts, wheat grass, barley grass, or broccoli [31].

If the first-line antioxidants act to suppress or prevent the formation of free radicals or reactive species in cells being very fast in neutralizing molecules with the potential of developing into a free radical or neutralizing any free radical with the ability to induce the production of other radicals [31], the second-line defense antioxidants (scavenging antioxidants) are neutralizing or scavenging free radicals by donating an electron to them, becoming free radicals themselves but of lesser damaging effects [31,82]. These are mainly represented by hydrophilic antioxidants, such as ascorbic acid, uric acid, and glutathione, and by lipophilic antioxidants, such as alpha-tocopherol (vitamin E) and ubiquinol [31,82].

After free radical damage has occurred, a third category of antioxidants (de novo enzymes), such as polymerases, glycosylases, nucleases, proteinases, proteases, and peptidases, are acting towards repairing the damage caused to biomolecules and reconstitute the damaged cell membrane [31], while a fourth-line defense antioxidants can prevent the formation or reaction of free radicals [31,82].

## 3. Antioxidants as Food Additives

Food additives that are approved for use in Europe are annotated with an E, which is followed by at least three digits. There are several categories of additives, one being antioxidants. According to the directives of the European Parliament and the Board of Directors 95/2EEC and 98/85/EEC, the antioxidants authorized to be used in food products are those between E-300 and E-321 and include a series of natural compounds (E-300, L-ascorbic acid; E-306, the natural extract rich in tocopherols) but especially synthetic compounds, with toxic potential [103]. Table 2 lists those antioxidants commonly used as food additives using the nomenclature of [6].

A wide range of antioxidants can be used to stabilize food products, but their use is limited by health regulations. The conditions that an antioxidant must fulfil to benefit from the legal permission for use in food products are the following [42,105,106,107,108]:

➢ The addition of the antioxidant must be authorized by the legislation of the country where the food products will be consumed.
➢ The action of the antioxidant must not be limited only to finding it as such and must be limited to food products in which the respective fat was later incorporated as an ingredient.
➢ The addition of the antioxidant must be simple, without lengthy or complicated manipulations.
➢ The appearance or taste of the respective product must not be modified in any way by the presence of the antioxidant.
➢ No negative effect on the human body is allowed even after continuous and prolonged incorporation in the daily food ration.
➢ The antioxidant must be effective in very small quantities so that its addition exerts an insignificant influence on the cost price of the respective product.
➢ The presence of the antioxidant in fats or other food products must be able to be determined through simple analysis, preferably both quantitatively and qualitatively.

Currently, the focus in industry and research is not only to find new antioxidants to further fulfil these legal conditions but also to replace as many synthetic food additives [109] as possible with ones based on natural antioxidants [110] from vegetable sources, and special attention is given to the ones of marine origin [111] for lipid systems. From the biochemical point of view, it is known that antioxidants can be for lipid systems or for hydrophilic systems [82,87,110], the ones for lipid systems are widely used in the food industry [109], and some examples are given below [86,109].

*Butylhydroxyanisole* (BHA) is composed of a mixture of two isomers 2 and 3 tributyl-4-hydroxyanisole (C_11_H_16_O_2_). It is a white-yellow crystalline substance insoluble in water, but soluble in ethyl alcohol and other organic solvents [112,113]. It has good resistance to high temperatures, and as a result, it can be used for frying, boiling, and baking products. It is carefully used in lower concentrations of 0.01%–0.02% for its antioxidant effect [112,113]. Despite its favorable properties, rational use of BHA must be considered [114], as at higher concentrations, it can cause carcinogenicity, cytotoxicity, oxidative stress induction, endocrine disruption [114,115], and important side effects of tert-butylhydroxyanisole, such as reducing hepatic enzymes or toxic effects in lung tissue [116].

*Butylhydroxytoluene* (C_15_H_24_O), also known as BHT, is presented in the form of white crystals or sequins, with a weak phenolic smell. It is insoluble in water, but soluble in alcohol [114]. A dose-related increased incidence of the severity of toxic nephrosis, nephrotoxicity, and pneumotoxicity, and marked congestion of the liver and kidney [114], as well as diffuse enlargement of the liver with rounded borders and rupture with hemorrhaging, were cited as toxic effects of BHT in mice [117,118] or in rats [119,120]. Fourteen metabolites or degradation products of BHT were at an increased level of concern about their toxic effects (BHT-CH_2_OH, BHT-CHO, BHT-COOH, BHT-Q, BHT-QM, DBP, BHT-OH, BHT-OOH, TBP, BHQ, BHT-OH(t), BHT-OH(t)QM, 2-BHT, and 2-BHT-QM), with reviewed effect on in vitro DNA cleavage for BHT-Q at the lowest concentration [121]; BHT-CHO and BHT-OOH were also cited with such ability to cause DNA cleavage, but not for BHT, BHT-CH_2_OH, BHT-COOH, and BHT-QM [121]. Mice-fed dietary BHT for a year were known to develop marked hyperplasia of the hepatic bile ducts with associated subacute cholangitis [117,118]. Dose-related increases in hepatocellular adenomas and carcinomas were also cited in the case of rats [119,120,121].

*Gallic acid esters* are widely used as antioxidants [122,123,124,125], being derivatives of a naturally occurring low-molecular-weight triphenolic compound, the gallic acid (3,4,5-trihydroxy-benzoic acid) has a strong antioxidant and an efficient apoptosis-inducing agent effects [125]. Some reports on their contact-sensitizing ability (using topically applied products) correlated with the side chain length were documented, while a maximum of sensitization occurrence was tested for 12 carbon atoms length of the molecule (dodecyl gallate) [126,127]. Currently, the use of long-chain gallic acid esters, such as octal and dodecyl gallates, is preferred [122,123,124,125,126,127], which have a much better distribution coefficient and are more effective for the protection of fat/water systems.

*Tocopherols* are widely distributed in nature, having the role of natural antioxidants, and being represented by vitamin E. Among the isomers of tocopherol, *δ*-tocopherol has the greatest effectiveness as an antioxidant [128,129]. It is presented in the form of viscous, yellowish oil [128]. Δ-tocopherol was reported as more active than *α*- or *γ*-tocopherol in inhibiting tumor growth, possibly through trapping reactive oxygen and nitrogen species and inducing apoptosis [130,131].

*Ascorbyl palmitate* is the ester of palmitic acid with ascorbic acid and is obtained by the synthesis of two components that are naturally found in food [132]. Concentrations of 0.01% ascorbyl palmitate [133] were reported to provide a useful increase in the shelf-life of vegetable oils [132], with a better action in this regard when compared to butylated hydroxytoluene and butylated hydroxyanisole [134]. Moreover, combinations with other known antioxidants were shown to improve the shelf-life of all vegetable oils, as well as potato chips [135,136].

Among the antioxidants for hydrophilic systems, especially for use in wines, juices, and fruit derivatives, two have found a wide application so far: SO_2_ and ascorbic acid [137]. The sulfur dioxide exerts a double effect as it inhibits oxidizing enzymes and at the same time has a strong reducing action [138]. It has been established that the oxidizing enzymes, polyphenoloxidases, peroxidases, and ascorbinoxidases are inhibited in the case of using large doses of sulfur dioxide, acting on the prosthetic groups found in the enzymes [138]. Sulfur dioxide has a bleaching effect, a process that is, however, irreversible. In human consumers, allergies caused by sulfites (SO_2_-derived compounds) were documented, including symptoms of their expression, such as headaches, nausea, gastric irritation, and breathing difficulties in asthma patients [139].

Ascorbic acid, due to its reducing properties, achieves the inhibition of oxidation processes in concentrations of 100–200 mg/L. It is used at lower concentrations than sulfur dioxide to avoid toxicity, since at higher concentrations the ascorbic acid was cited to act as an antioxidant, while at lower concentrations, as a pro-oxidant [140,141]. Although the combined use of ascorbic acid and sulfur dioxide was initially assumed as with many advantages over the use of either compound alone, later reviewed studies suggested that ascorbic acid may not be the ideal complement to sulfur dioxide as first considered [142].

Along with antioxidants, in the food industry, synergistic substances are also used, which do not have antioxidant action, but promote it, including citric acid, phosphoric acid, and ethylenediaminetetraacetic acid (EDTA) [143,144,145].

There are also those known as antioxidant combinations meant to ensure an optimal effect, in which mixtures of two or more antioxidants are used, associated with synergists, ensuring optimal product stability [143,144,145]. Thus, for animal fats, a solution containing 20% BHA, 6% propellant gallate, and 4% citric acid is used. French fries and fries with a high fat or oil content can be very well protected by a mixture of BHA, BHT, propellant gallate, and citric acid. In the case of meat dishes, the mixture of BHA and citric acid works best. Antioxidants can also be used to extend the shelf life of frozen fish, preventing the appearance of yellow-brown color due to the oxidation and polymerization of fats [110].

As industry tends to shift from synthetically produced preservatives to natural preservatives, more and more studies are established in this direction, the interest being to lower the unnecessary chemical burden on health and to naturally prevent food degradation. There are reports showing that natural antioxidants from fruits (grapes, pomegranate, date, kinnow, plums, avocado, and tomato), herbs and spices (tea, rosemary, oregano, cinnamon, sage, thyme, mint, ginger, and clove), vegetables extract from broccoli, potatoes, drumstick, pumpkin, curry, and nettle are used as additional additives or antioxidants in food preservation [146,147].

Due to their high content of phenolic compounds, they provide alternatives to currently used conventional antioxidants, prolonging the shelf life of foods [146,147]. For example, in the meat industry, these natural antioxidant extracts are used for improving the quality of fresh and processed meat and meat products by decreasing lipid oxidation [146,148,149,150,151]. Other examples are the use of terpenoids and polyphenols in the prevention of lipid oxidation of meat, fish, or vegetable food with different amounts of saturated/polyunsaturated fat for ensuring high sensorial quality and food preservation [107,152]. Moreover, it was shown that powdered leaves of matcha green tea and moringa added to white chocolate during the tempering process improved its antioxidant capacity due to the high amounts of polyphenols in green tea [146,153]. Another recent study showed that lemongrass essential oil has a higher applicability as a food preservative due to its content in terpenes [154]. From natural marine algae, seaweeds are known to have several properties to act as natural preservatives extending the shelf life of perishable foods and at the same time not affecting their quality or causing side effects. An applicable example is the seaweed gel coating used to protect tomatoes from perishing [155].

On the other hand, active food packaging containing natural antioxidants, such as α-tocopherol, caffeic acid, catechin, quercetin, carvacrol, and plant extracts (e.g., rosemary extract), has been implemented in recent years as there is more advantage than the addition of antioxidants directly to the food [156]. One important interest is in active biodegradable/compostable packaging and edible films to reduce environmental impact, minimize food loss and contaminants from industrial production, and reutilize by-products [156], active packaging being biocompatible and eco-friendly [157], having also other properties, such as antimicrobial, antioxidant, UV blocking, oxygen scavenging, and water vapor permeability effects [158].

## 4. Natural Antioxidants and Their Benefits

In addition to established antioxidants, which fall into the category of additives, there are natural antioxidants that can be obtained through extraction from different plants [86]. Extracts from rosemary, aloe vera, fenugreek (*Trigonella foenum*), ginseng, mustard, sage, oregano, horseradish, hyssop, basil, marjoram, mint, thyme, ginger, cumin, cloves, nutmeg, curry, cinnamon, black pepper, green tea, coffee, grape skin and seeds, and pine bark have been used with various food groups [86], giving equivalent or better results than those obtained with synthetic antioxidants (BHA, BHT, and gallates). However, the use of these extracts is limited by the intensity of their flavor which influences the taste and smell of the products to which they are added. Essential antioxidants include vitamins A, C, E, and beta-carotene, which are helpful in slowing down the aging process and protecting the body from cancer, heart disease, and pollution. They also help strengthen the immune system and increase the body’s resistance to infections. Every year, more and more antioxidants are discovered [110,111,159]. Among them, there are substances from forest fruits, grapes, tomatoes, broccoli, and mustard, as well as those from medicinal plants, such as turmeric and *Ginkgo Biloba* but also algae extract. In addition to the already well-known antioxidant role of vitamins A, C, and E, there are other important antioxidants, including lipoic acid, carnitine, lutein, and lycopene [160,161,162], and currently, there is still a high interest in discovering novel antioxidants from fruits and vegetables as they are a main source of antioxidants, known by their antioxidant expression values.

One way of expressing the antioxidant values of food products is in ORAC units (the oxygen radical absorption capacity expressed in units), a unit of measure approved by the National Institute for the Elderly within the National Institute of Health (INS) [163]. An ORAC unit is expressed in micromoles of Trolox equivalents (TE) per 100 g of sample. The ORAC scale presents in ascending order the antioxidant values that foods have up to the present time. Recently, an evaluation of antioxidant capacity (ABTS and CUPRAC) and total phenolic content (Folin–Ciocalteu) assays of selected fruits, vegetables, and spices has also been established [164,165]. While the ORAC value is a method of measuring the antioxidant potential of different food products and supplements [163], these recent scales highlight the common foods that have increased antioxidant effects.

One ORAC unit destroys free radicals in addition to the fact that our body naturally destroys free radicals only through its production of antioxidant enzymes. FDA’s recommendation of ORAC units per day administration is the amount between 3000 and 5000 µmol TE per day (ORAC) from plant foods, especially in the presence of constant pro-oxidant factors [166] but has been shown that ≥10,000 µmol TE per day (ORAC) has positive health effects [163]. This amount is necessary for the prevention of various diseases and to maintain health. Moreover, in the situations of people with diseases, such as degenerative diseases, acute diseases, cancer, lymphomas, leukemias, and AIDS, a higher intake of ORAC units/day is recommended [163,166].

In order to inform about the total antioxidant content of foods, there is an available database that comprises antioxidant food content [167], based on several assays, such as 6-hydroxy-2,5,7,8-tetramethylchroman-2-carboxylic acid (Trolox-equivalent antioxidant capacity (TEAC)) assay, the ferric-reducing ability of plasma (FRAP), and the oxygen radical absorbance capacity assay (ORAC) [168].

### 4.1. Food and Fruits Antioxidants

Various studies on nutritional issues have revealed that a diet rich in fruits and vegetables is important due to their sources of nutrients and nonnutritive food constituents, showing that a high daily intake of fruits and vegetables promotes health [169]. In the meantime, it is known that low fruit and vegetable consumption is linked with an increased risk of death from vascular disease and cancer; these benefits are attributed in part to antioxidants, vitamins, and phytochemicals [170]. Although phytotherapeutic substances and antioxidants exist in their purest form in fruits, vegetables, and cereals, a balance of the daily calories’ intake must be considered as well, as to metabolize calories, the body burns oxygen, generating free radicals [12,171,172]; hence, the more calories are consumed, more free radicals are generated. A balance in the antioxidant uptake, as well as certain pathological conditions such as cancer, must be considered since there are studies showing risks of an imbalanced antioxidant uptake for cancer patients, since both excess and lack of antioxidants can affect and negatively influence the normal cellular processes [173,174,175].

There are over 2000 phytotherapeutic substances contained in food, and these are defined as natural compounds that act as a plant defense system [176]. Various types of food are rich in antioxidants and phytotherapeutic substances such as: 

*Tomatoes*—One medium tomato contains 26 calories and 0 g of fat. Biochemically speaking, tomatoes are rich in lycopene and are a great source of vitamin C [176,177].

*Spinach*—100 g of spinach contains 41 calories and 0 g of fat and is rich in iron, folic acid, and B vitamins [178]. In addition, spinach contains two phytotherapeutic substances, lutein and zeaxanthin, which are very important for eye health [176,178]. It is recommended to be eaten, as much as possible, raw or scalded [178].

*Nuts*—30 g of nuts contain 12 g of fat and 150 calories. Although they are high in fat, walnuts contain good monosaturated and polyunsaturated fats [179]. When eaten in place of red meat or high-fat foods, walnuts help lower bad cholesterol (LDL) and increase good cholesterol (HDL) [176,179]. Nuts also contain a well-known antioxidant, vitamin E. However, they contain higher calories, and therefore, their consumption is indicated in limited quantities [176,179].

*Broccoli*—100 g of cooked broccoli contains 44 calories and 0 g of fat. Broccoli is an important source of vitamin C, fiber, and calcium [180,181]. This vegetable also provides phytotherapeutic substances used as natural antibiotics, antiviral drugs, and antimycotics, contributing to protection from DNA damage and preventing the formation of cancer cells [176,180,181]. The deep thermal processing of this vegetable destroys the enzymes and the corresponding nutrients and therefore it is recommended to consume it fresh or just scalded [176].

*Blueberries*—100 g of blueberries contain 81 calories and 0 g of fat and represents a significant source of antioxidants [176,182]. Anthocyanin is the phytotherapeutic substance that gives the dark blue color, specific to blueberries, which prevents the formation of cancer cells and has a curative effect on urinary infections [182]. A regular, moderate intake of blueberries and/or anthocyanins was associated with a reduced risk of cardiovascular disease, and type 2 diabetes, and with improved weight maintenance and neuroprotection [182,183].

*Green tea*—In Asia, green tea is consumed in the same amount as coffee in the Western world [176]. Studies correlate the consumption of green tea with the low incidence of stomach, esophagus, and liver cancers [176,184,185,186,187]. Polyphenol, the phytotherapeutic substance in green tea, has been identified as an anticancer agent [184,185,186,187].

Adding to the above examples, Table 3 also displays other foods containing important antioxidants [168,188,189,190].

*Anthocyanins* are part of the phenolic group with the pigments (red, purple, and blue) in glycosylated forms and are found in black rice, blackberries, blueberries, eggplant, grapes, and raspberry [191,192] (Table 3) with the most abundant pigment in plants, the cyanidin-3-glucoside. Apart from being used as a natural food colorant, anthocyanins are considered pharmaceutical ingredients that give various beneficial health effects, being reported as having antidiabetic, anticancer, anti-inflammatory, antimicrobial, and antiobesity effects [192,193,194].

*Beta-carotene* is part of the carotenoid family. It reaches our body partially transformed into vitamin A, found mainly in colorful vegetables and fruits [195,196] (Table 3). *Β*-carotene administered orally was reported to be metabolized in the animal or human body to form vitamin A, which is subsequently stored in the liver [195,196].

*Catechins* are flavanols found in wine, green tea, and cocoa [197,198], which have potent antioxidant properties, in the meantime being known as reactive oxygen species (ROS) scavengers and metal ion chelators. They have indirect antioxidant activities comprising induction of antioxidant enzymes, inhibition of pro-oxidant enzymes, production of detoxification enzymes and antioxidant enzymes. For these reasons, they are being considered beneficial in preventing and protecting against diseases caused by oxidative stress [197,198].

*Cryptoxanthines* are found in butter, citrus, egg yolk, papaya, red bell peppers, and pumpkins [199,200], having relatively high bioavailability from these natural resources [199]. Among other carotenoids, β-cryptoxanthin has high antioxidant activity and promotes free radical scavenging, protecting against chronic diseases [200].

*Copper* is found in dark chocolate, liver, lobster, oysters, spirulina, and shiitake mushrooms [201,202], and it was shown that dietary copper could improve antioxidant capacity and immune state [202].

*Flavonoids* are abundant in broccoli, citrus, kale, parsley, onions, strawberries, and tea [203,204,205,206], and their antioxidant capacity has been intensely proven in the last years [203,204,205,206]. They are a large group of diverse polyphenolic compounds of plant origin classified into major classes, including flavonols, flavones, flavanones, flavanols, anthocyanidins, isoflavones, and chalcones [206], and nowadays are considered an indispensable component in a variety of medicinal, pharmaceutical, nutraceutical, and cosmetic preparations [203,204,205,206].

*Indoles* are found in broccoli, cabbage, cauliflower, mustard seeds, and turnips [207,208]. They are known for their chemopreventive effects on hormone-dependent cancers and inhibit proliferation, migration, and invasion of cancer cells in vitro studies [207,208].

*Isoflavonoids* can be found in chickpeas, peanuts, pistachio, and soybeans [209,210], and they are very well known for their therapeutic properties, having anti-inflammatory, estrogenic, antiestrogenic, anticancer, antibiotic, and radical scavenging activities [209] and being involved as well in the prevention of cancer [210].

*Lignans*, in barley, flaxseed, rye, and sesame seeds [211,212], are used as pharmacological agents in disorders related to oxidative stress and inflammation [211], as they possess a strong anti-inflammatory and antioxidant capacity [211,212].

*Lutein* is one of the few xanthophyll carotenoids that is found in high concentration in the macula of the human retina, and as it cannot be de novo synthesis within the human body, lutein must be obtained from natural food, such as kale, and parsley, peas, spinach, and tomatoes [213,214].

**Figure 6:**
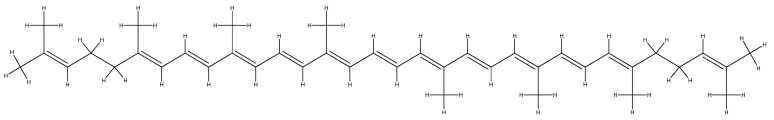
The molecular structure of lycopene.

*Lycopene* is a carotenoid contained especially in tomatoes, with antioxidant and detoxifying properties. It helps the proper functioning of the cell growth process and has a beneficial influence on the skin and mucous membranes [215,216]. Studies show that lycopene inhibits the development of esophageal, stomach, colon, breast, and prostate cancer cells [215,216]. The antioxidant action of carotenoids includes their ability to capture singlet oxygen, an action possible due to their chemical structure (Figure 6); lycopene, for example, is the most effective of carotenoids in capturing the reactive oxygen species [215,216].

*Manganese* is found in acai berries, almonds, brown rice, pecans, pineapples, pinto beans, and whole wheat bread [217,218]. Due to its component integration for Mn superoxide dismutase (MnSOD), it is mainly responsible for scavenging reactive oxygen species (ROS) in mitochondrial oxidative stress [217]. Avoiding its deficiency and intoxication is essential as otherwise, the imbalance can lead to associated adverse metabolic and neuropsychiatric effects [217,218].

*Polyphenols* stimulate the body’s natural defense capacity and protect human cells from oxidative stress [219,220]. They prevent skin aging and degenerative diseases, such as cardiovascular diseases, and osteoporosis [219] and have anticarcinogenic properties by suppressing tumor formation and progression [221,222,223]. Polyphenols, such as quercetin, resveratrol, and curcumin, are more potent antioxidants than vitamins C and E, having a faster antioxidant action [224,225], and enhancing the therapeutic profiles [226]. Polyphenols are represented by several thousand plant-based molecules with antioxidant properties [227], classified into flavonoids (i.e., anthocyanins, flavanols, flavanones, flavonols, flavonones, flavones, and isoflavones) and non-flavonoids (i.e., phenolic acids, xanthones, stilbens, lignans, and tannins), and known for their high potential application in food preservation and for therapeutic beneficial use [227].

*Selenium* is the main mineral antioxidant and deactivator of free radicals, and it is effective in preventing degenerative diseases, including cancer and cardiovascular diseases [228,229]. As an antioxidant, selenium acts by intervening in the activation of glutathione peroxidase [230], an enzyme with a role in the detoxification of lipid and organic peroxides in the cell, thus preventing alterations caused by peroxidation of cellular macromolecules [230]. Another action of selenium is influencing the activity of DNA polymerase and nucleotide kinases or inducing the synthesis of selenoproteins [231,232]. Depending on the dosage and chemical form of selenium and the nature of the carcinogenic stress, selenium is proposed as an anticarcinogenic agent, owing, among other factors, to reversible or irreversible inhibition of protein and DNA synthesis [230]. Selenium is an essential mineral for both humans and animals, found in all types of soil around the world [228,229]. Plants and small animal organisms convert selenium found in the soil into organic components, such as seleno-methionine or chelated selenium, the form in which selenium is found in food [233,234]. It binds to proteins and is absorbed into the body in its original form without undergoing metabolic changes [233,234]. The human body needs this mineral in a small amount, as a too high level can have toxic effects on the body, so the recommended daily allowance of selenium is 55 mcg/day for women and 70 mcg/day for men, and the tolerable upper limit is 400 mcg/day, while the deficiency is defined as less than 30 mcg/day [235]. Selenium deficiency has been reported quite rarely, and the need for selenium supplements is not necessary and is available only by prescription in some cases [233,234].

*Sulphur* is a structural component in chives, garlic, leeks, and onions [236,237]. It is an important element in biological systems as being integrated into proteins as the redox-active cysteine residue or in vital antioxidant molecules, such as glutathione, thioredoxin, and glutaredoxin [236,237].

*Vitamin A* or all-trans-retinal, which accumulates in the retina because of the absorption of light by visual pigments, is found in carrots, egg yolk, liver, and sweet potatoes [238,239]. Being a liposoluble vitamin, a balance in its intake is to be considered [239].

**Figure 7:**
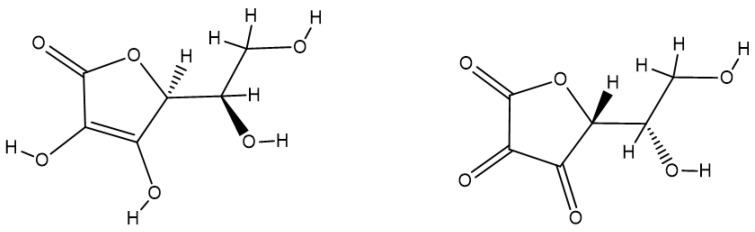
The molecular structure of ascorbic acid (**left**) and dehydroascorbic acid (**right**).

*Vitamin C* or L-ascorbic acid is essential in maintaining health as it prevents and fights infections, promotes wound healing, and prevents stress and fatigue [224,225]. Vitamin C is also involved in skin health and protection, preventing the appearance of wrinkles as well [240]. Vitamin C is a water-soluble vitamin and is also considered one of the most important antioxidants, acting at the level of extracellular fluids [224,225]. Due to its properties, its lack in the body leads to a drastic decrease in the immune system in the fight against infections [241]. In contrast to most animals, the human body cannot synthesize vitamin C, because of a mutation in the last enzyme required for ascorbate biosynthesis [58,242]. Thus, vitamin C must be obtained from our daily diet [241]. The oxidized form of ascorbic acid is represented by dehydroascorbic acid (Figure 7), and the regeneration of ascorbate from this oxidized form is necessary to maintain sufficient tissue levels of the reduced form of vitamin C [243].

**Figure 8:**
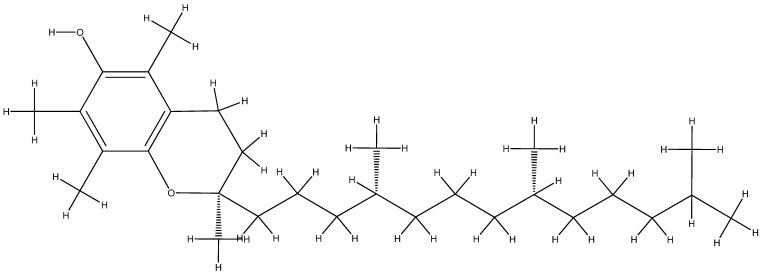
The molecular structure of vitamin E.

*Vitamin E* is one of the best natural antiaging remedies, comprising eight forms of tocopherols and tocotrienols [224,225]. Its action is particularly related to the genital sphere, having an important role in the fecundity mechanism [244], stimulating cell regeneration, protecting cells and tissues from the action of free radicals, and having a therapeutic role in cancer [245]. Depending on the number and position of the methyl groups attached to the chromanol ring, they are named by joining the Latin letters *α*, *β*, *γ*, and *δ*; *α*-tocopherol (Figure 8) and *γ*-tocopherol being the most important forms of vitamin E [246] are found in vegetable oils, sunflower seeds, vegetables, and fruits [240]. Soybean oil contains a mixture of *γ*, *δ*, and *α*-tocopherol, with a cited tocopherol content in corn oil and soybean oil of 77% and 70% *γ*-tocopherol, 2% and 23% *δ*-tocopherol, and 14% and 7% of *α*-tocopherol, respectively [247].

*Zinc* is an important mineral as the human body cannot store zinc reserves, so this needs to be added to the diet, especially because zinc is influencing the immune system, transcription factors, cell differentiation and proliferation, DNA and RNA synthesis and repair, enzyme activation or inhibition, the regulation of cellular signaling, and the stabilization of the cell structure and membranes [248,249]. Avocados, chicken, oatmeal, pork, and Shiitake mushrooms present higher amounts of zinc [248,249].

**Figure 9:**
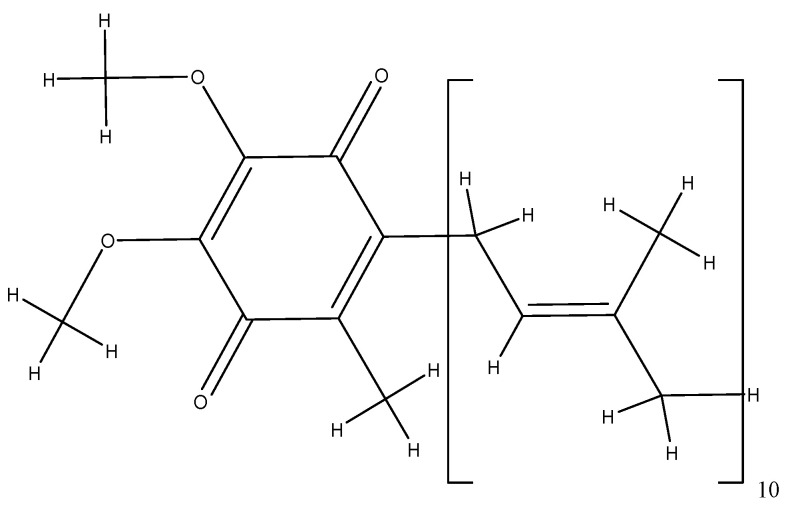
The molecular structure of coenzyme Q10.

*Coenzyme Q10* is a fat-soluble antioxidant that ensures the transport of oxygen inside the cells and the production of energy becoming amphiphilic following electron and proton interactions [250]. Q10 represents its molecular structure, made up of 10 isoprene units around the quinone ring, and the number of isoprene units in the side chain varies with the species (Figure 9) [250,251]. Coenzyme Q10 is a natural compound, which is produced in the body following the consumption of fish, sardines, nuts, green vegetables, soybeans, and oilseeds [252]. It is a good ally against skin aging [253], and it is effective in preventing and treating cardiovascular diseases, especially heart rhythm disorders [254,255], and considerably reduces the risk of breast, uterine, lung, and colon cancers [256,257,258,259,260]. In addition to the antioxidant function, coenzyme Q10 has the capacity to donate electrons, contributing to the production of bioenergy by oxidizing glucose in the mitochondria, where the free radicals are produced during the process of cellular respiration [89,90,91,92,261].

The plant-based diet has become the equivalent of many curative and prophylactic treatments [48,109,262,263]. The importance of the consumption of vegetables and fruits is explained by the beneficial intake of antioxidants as they are being active in numerous biological processes of the body at the cellular level [109,264]. Moreover, most studies [265,266,267] indicate that animal foods should be substantially reduced and replaced with fresh, minimally processed plant foods to reduce the prevalence of cancer.

### 4.2. Marine Algae Antioxidants

**Figure 10:**
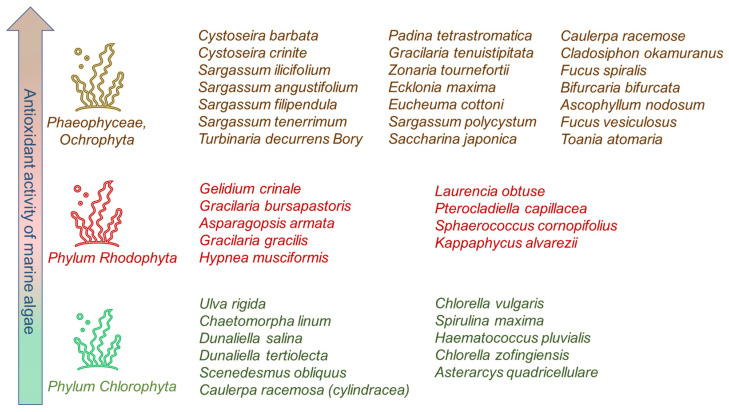
Antioxidant activity of marine algae and representative species, with higher antioxidant activity range from brown algae to green algae. This figure is based on information from references [270,274,275,278].

**Figure 11:**
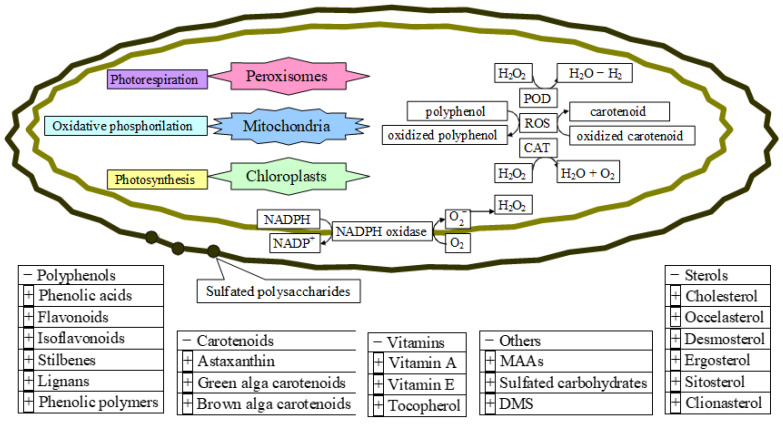
The microalgae antioxidants and their mechanism of action at the cellular level. POD, peroxidase; CAT, catalase.

Considerable attention must be paid to the algae antioxidants as many recent studies showed their high beneficial potential and importance (Figure 10 and Figure 11) [268,269].

A classification of marine macroalgae is known as brown algae (*Phaeophyceae*), red algae (*Phylum Rhodophyta*), and green algae (*Phylum Chlorophyta*), according to their pigmentation [270,271,272]. Representative species of brown, red, and green algae with higher antioxidant activity are presented in Figure 10. Marine antioxidants from seaweeds have a very high antioxidant potency, currently being known as the mechanisms of antioxidative action for at least 301 macroalgal metabolites [270,273].

As shown in Figure 10, the trend of antioxidant potential is going from the highest antioxidant activity of brown algae extract followed by red and green algae extract as shown in several studies [270,274,275]. Their high antioxidant activity is probably due to the synergetic coexistence of polyphenols and alkaloids [276,277].

The future of marine algae extracts providing resources as food antioxidants additives is very promising since they are containing compounds from polyphenols to carotenoids, sterols, vitamins, and several others from at least 50,000 species of known marine microalgal classes [273,279,280,281] (Figure 11). Moreover, the favorable biological activities of microalgae are due to their intrinsic antioxidant, anti-inflammatory, and antitumoral features [279,282].

Their intrinsic mechanism of taking up H_2_O and CO_2_ combined with the sunlight, and converting them to complex organic compounds, makes them subsequently kept inside or released from the cell. Figure 11 represents the biological background of antioxidant formation in microalgae as a response to oxidative stress, at the cellular level, listing the main antioxidant compounds found in microalgae [270,273,279,280,281,282,283,284,285,286,287].

**Figure 12:**
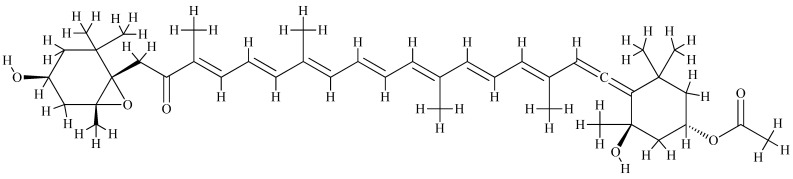
The molecular structure of fucoxanthin (cis).

*Fucoxanthin,* for example, has a particularly interesting and unique molecular structure, exhibiting antioxidant properties due to a long-conjugated backbone characteristic of all carotenoids (Figure 12) [288], but possessing an unusual terminal allenic bond and conjugated carbonyl groups [289]. Fucoxanthin is a member of the xanthophyll class of carotenoids and is present at high concentrations in the brown algae *Saccharina* sp. [288,289,290,291], where it plays a key role in light harvesting and radiation protection [291].

**Figure 13:**
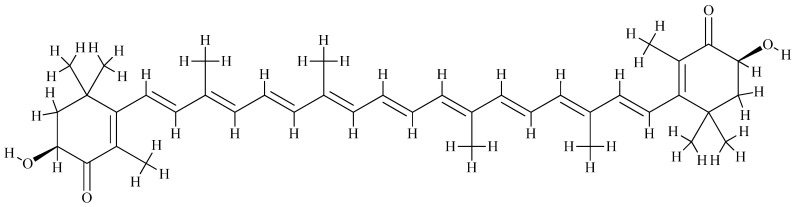
The molecular structure of astaxanthin (trans).

*Astaxanthin* (Figure 13) is another fat-soluble xanthophyll carotenoid with a red pigmentation, which is found in various microorganisms and marine animals and algae [292,293] such as *Haematococcus pluvialis*, a green microalga, which accumulates high astaxanthin amounts under stress conditions of high salinity, nitrogen deficiency, high temperature, and light [294,295,296]. In a few years, it has been approved to be used as a food colorant in animal and fish feed such as salmon, trout, and shrimp [292,297] and used as a nutritional supplement and has been rapidly growing in foods, feeds, nutraceuticals, and pharmaceuticals [298,299].

Other potential resources of marine macroalgae components known to exhibit significant antioxidant activities are marine secondary metabolites such as bromophenols (containing one or several phenols with one or more bromine atoms), present in all three algae types, red, brown, and green algae, phlorotannins [273,300], terpenoids, and meroterpenoids [301], which are to be further investigated.

## 5. Conclusions

From endogenous to exogenous antioxidants mentioned in this review, we have outlined the special importance of the presence of antioxidants in food, fruits, and their consumption, as well as their use as food additives.

Given that over years, some food additives proved to have carcinogenic effects or to cause other health issues, exploring the use of alternative antioxidants in the food industry became a high priority.

Understanding the biochemical defense mechanisms against free radicals and the mechanisms of antioxidant activity, altogether with the intracellular antioxidant balance, is complex as has been illustrated in this review.

Marine resources of possible new food antioxidants are of much current interest due to their high biodiversity as well as their ability to adapt and colonize very different types of aquatic ecosystems. Further exploration of their properties via testing the antioxidant activity in vitro, and in vivo, including economic and environmental concerns and possible negative side effects such as toxicological issues is hence warranted.

## Figures and Tables

**Table 1:** Inhibitors of lipid oxidation reactions.

Type	Mode of Action	Examples
“Metal scavenger”	Chelates metal ions such as copper and iron, forming inactive complexes	Chelating agents such as EDTA, citric acid, phospholipids, polyphosphates
“Oxygen scavenger”	Reacts with oxygen; reduces oxygen	Ascorbic acid, ascorbyl-palmitate
Antioxidant (AH)	Interrupts propagation stages in the case of oxidation reactions; donates a hydrogen atom	Phenolic compounds such as BHA, BHT, TBHQ, PG, tocopherols, hydroxytyrosol, caffeic acid, carnosol, etc.
Reducing agents (RSH)	Regenerates phenols (synergism)	Ascorbic acid
Enzymatic antioxidant	Removes dissolved oxygen or oxidative species	Superoxide dismutase, glutathione peroxidase, glucose-oxidase-catalase
Antioxidants with multiple functions	Regenerates primary antioxidants chelated with metals; reduces hydroperoxides	Phospholipids (phosphatidyl-ethanol amine- fish oil), products of the Maillard reaction
Methyl-silicone and ethylidene phytosterols	They prevent oxidative polymerization in heated oils	Polydimethylsiloxane, citrostadienol

**Table 2:** Inhibitors of lipid oxidation reactions.

Annotation	Antioxidant	Annotation	Antioxidant
E300	Ascorbic acid	E310	Propyl gallate
E301	Sodium ascorbate	E315	Erythorbic acid
E302	Calcium ascorbate	E316	Sodium erythorbate
E304	Fatty acid esters of ascorbic acid	E319	Tertiary-butyl hydroquinone
			(TBHQ)
E306	Tocopherols	E320	Butylated hydroxyanisole
			(BHA)
E307	*α*-tocopherol	E321	Butylated hydroxytoluene
			(BHT)
E308	*γ*-tocopherol	E392	Extracts of rosemary
E309	*δ*-tocopherol	E586	4-Hexylresorcinol

**Table 3:** Antioxidants in foods.

	Anthocyanins	β-Carotene	Catechins	Cryptoxanthines	Copper	Flavonoids	Indoles	Isoflavonoides	Lignans	Lutein	Lycopene	Manganese	Polyphenols	Selenium	Sulphur	Vit. A	Vit. C	Vit. E	Zinc
Acai berries												✔							
Almonds												✔							
Apricots		✔																	
Artichokes													✔						
Avocados																		✔	✔
Barley									✔										
Black currants																	✔		
Black rice	✔																		
Blackberries	✔																		
Blueberries	✔																		
Brazil nuts														✔					
Broccoli							✔										✔		
Brown rice												✔							
Brussels sprouts						✔													
Butter				✔															
Cabbage							✔												
Carrots		✔														✔			
Cauliflower							✔										✔		
Cherries													✔						
Chicken																			✔
Chickpeas								✔											
Chives															✔				
Citrus				✔		✔											✔		
Cocoa			✔																
Coffee													✔						
Cottage cheese														✔					
Dark chocolate					✔														
Egg yolk				✔												✔			
Eggplant	✔																		
Eggs														✔					
Extra virgin olive oil																		✔	
Flaxseed									✔										
Garlic															✔				
Grapefruit											✔								
Grapes	✔																		
Green tea			✔																
Kale						✔				✔							✔		
Kiwi																		✔	
Leeks															✔				
Lentils														✔					
Liver					✔											✔			
Lobster					✔														
Mangoes		✔															✔		
Mushrooms														✔					
Mustard seed							✔												
Oatmeal																			✔
Onions						✔									✔				
Oregano													✔						
Oysters					✔														
Papaya				✔							✔								
Parsley		✔				✔				✔									
Peanuts								✔											
Peas										✔									
Pecans												✔							
Pineapples												✔							
Pinto beans												✔							
Pistachios								✔											
Pork														✔					✔
Pumpkins				✔															
Raspberries	✔																		
Red bell peppers				✔															
Red grapes													✔						
Rye									✔										
Sardines														✔					
Sesame seeds									✔										
Shiitake mushrooms					✔														✔
Shrimp																		✔	
Soybeans *								✔											
Spinach										✔							✔		
Spirulina					✔														
Squash																		✔	
Strawberries						✔													
Sweet potatoes		✔														✔			
Tea						✔													
Thyme													✔						
Tomatoes										✔									
Turnips							✔												
Watermelon											✔								
Whole wheat bread												✔							
Wine			✔																

* Soybeans include Tofu, Edamame, and Tempeh. ✔ indicates the presence of the compound.

## Data Availability

The data is contained within the article.
